# “Ask a Doctor About Coronavirus”: How Physicians on Social Media Can Provide Valid Health Information During a Pandemic

**DOI:** 10.2196/24586

**Published:** 2021-04-20

**Authors:** Dorthe Furstrand, Andreas Pihl, Elif Bayram Orbe, Natasja Kingod, Jens Søndergaard

**Affiliations:** 1 Section for Health Services Research Department of Public Health University of Copenhagen Copenhagen Denmark; 2 Roche Diagnostics Copenhagen Denmark; 3 The Research Unit of General Practice Department of Public Health University of Southern Denmark Odense Denmark; 4 Department of Internal Medicine Herlev University Hospital Herlev Denmark; 5 Steno Diabetes Center Copenhagen Copenhagen Denmark

**Keywords:** COVID-19, coronavirus, digital health literacy, eHealth literacy, Facebook, framework, health information, health literacy, health promotion, infodemic, infodemiology, mental health, misinformation, pandemic, patient-physician relationship, public health, social media, trust, web-based community

## Abstract

In the wake of the COVID-19 pandemic, the information stream has overflowed with accurate information, misinformation, and constantly changing guidelines. There is a great need for guidance on the identification of trustworthy health information, and official channels are struggling to keep pace with this infodemic. Consequently, a Facebook group was created where volunteer medical physicians would answer laypeople’s questions about the 2019 novel coronavirus. There is not much precedence in health care professional–driven Facebook groups, and the framework was thus developed continuously. We ended up with an approach without room for debate, which fostered a sense of calmness, trust, and safety among the questioners. Substantial moderator effort was needed to ensure high quality and consistency through collaboration among the presently >200 physicians participating in this group. At the time of writing, the group provides a much-needed service to >58,000 people in Denmark during this crisis.

## Introduction

The world is currently facing a COVID-19 infodemic, and the immense information load complicates the identification of trustworthy health information [1]. The substantial growth of media sources has resulted in a dilution of relevant and reliable information. Because of the novelty and worldwide spread of COVID-19, we learn more about the virus every day and witness almost daily changes to the clinical recommendations. This is confusing for the public and for health care professionals. In April 2020, Limaye et al [2] called for health care professionals to accommodate this issue by building trust on social media. This viewpoint describes a novel eHealth literacy project in Denmark, where we have built a network of >200 volunteer medical physicians who provide up-to-date knowledge to a broad audience in a Facebook group with thousands of members.

## Health Information on Social Media

The Internet has become the primary source of health information for many people despite difficulties in verifying the reliability of web-based clinical evidence [3,4]. The emergence of Web 2.0 has further reoriented advancements from top-down information distribution to platforms for collaboration, dialogue, and content-sharing. One of the most significant components of Web 2.0 is social media platforms such as Facebook with approximately 2.4 billion users [5]. Since Facebook launched its community pages function in 2010, it became possible for people to create health-related groups, which have become larger interactive communities [6]. Web-based communities provide a space for social support, sharing of experiential data, and a collective voice [7]. Layperson-friendly explanations of medical terms help patients have more positive experiences when visiting their health care providers, thus improving the physician-patient relationship [8]. However, negative reactions from health care professionals toward patients’ social media activity might negatively affect patient-physician symmetries [9]; this underlines the need for health care professionals to engage on social media platforms to facilitate a positive physician-patient dynamic.

Owing to the COVID-19 pandemic, many new communities have been established on Facebook; however, this is accompanied with the risk of large-scale sharing of misinformation. The bombardment of emotionally evoking information makes it difficult for individuals to distinguish between accurate information and misinformation [10]. Although Facebook is mainly a lay-driven platform, health institutions, patients’ societies, and health care professionals are increasingly creating their own groups to interact with patients on the internet. As this is a rather new phenomenon, few studies have investigated the effect of Facebook groups created and moderated by health care professionals. Initial experiences indicate difficulties in establishing, disseminating, and scaling such networks on Facebook [11].

## The Facebook Group

**Figure 1:**
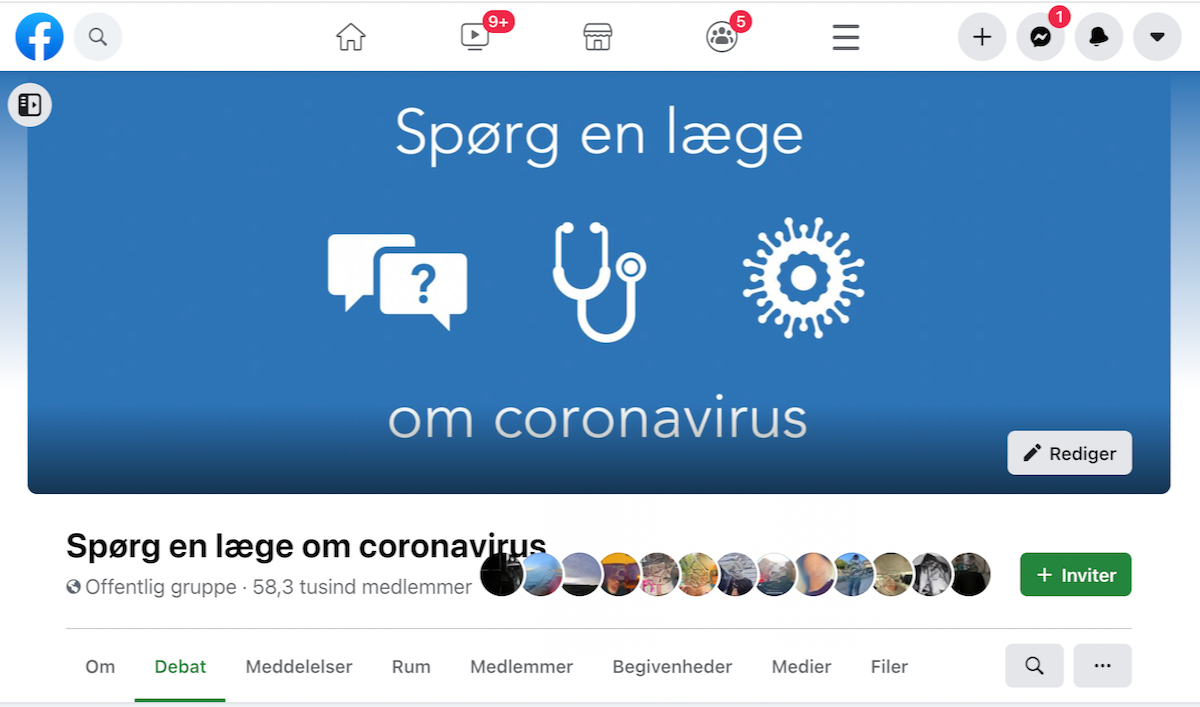
A screenshot from the Facebook group, “Spørg en læge om coronavirus,” which literally translates to “Ask a doctor about coronavirus.”.

In response to the infodemic, the Facebook group “Spørg en Læge om Coronavirus,” which literally translates to “Ask a Doctor About Coronavirus,” was created on March 15, 2020, by authors EBO and AP (Figure 1) [12]. This group was conceived owing to the sudden rise in waiting times on acute-care telehealth services. The continuously expanding group contains 57,000 members (approximately 1% of the entire population of Denmark) at the time of writing.

The rules of the group are conservative. Group members can post a question, and physicians with administrator privileges can either reject or accept the request and answer the question. Questions are of different types, spanning from basic knowledge on, for example, the biological, statistical, and epidemiologic aspects of the practical applications of the proposed guidelines to more complex existential questions on fear, the future, and hope. Approximately 30% of the incoming questions are rejected because they violate the group rules, such as those on politically motivated comments, weblinks to misinformation, and personalized health information. Rejections to requests are typically accompanied by an explanation. In parallel to the main group, where laypeople ask questions and physicians provide answers, we have a closed administrative physicians-only group to debate and share guidelines, news, and warnings against popular myths.

Running a group with >200 volunteer physicians takes active management. Early on, we realized that rules and structure were essential for all member physicians to feel safe. We selected a narrow approach without room for discussion related to the answers; when a physician has answered a question, the thread is closed for further commenting and unauthorized answers are deleted. This approach was selected owing to logistic reasons: it would be too time consuming for physicians to ensure the validity and relevance of the information posted freely in a thread. Surprisingly, the response from the users has been overwhelmingly positive. The calm and safe nature of the short threads of only physician-validated answers has proven a valuable factor, indicating the need for authoritative sources as a supplement to existing peer groups on Facebook. Unfortunately, the very rigid format of the group limits dialogue with the questioner. Furthermore, the practice has caused accusations of censorship. In the second wave of the COVID-19 pandemic, an increasing number of coronavirus skeptic members, verified through simultaneous membership in known government-critical or coronavirus skeptic groups, have joined the group and accused the group of censoring critical voices when closing threads and rejecting questions violating the rules; specifically those questions that are rejected because they contain weblinks to misinformation.

The ongoing stream of new questions provides us with a fine-tuned barometer of trending topics including circulating misinformation. Insecure members ask the group about the reliability of these topics before official information channels respond to the misinformation. We noticed that the users are highly perceptive to any inconsistency in the answers from different physicians, and we quickly identify any disruption in the sense of safety and security among the users. Fear and insecurity are directly articulated in the questions and comments, which confirms that feeling safe and in control is key to being eHealth-literate [13].

Meeting this need with consistency, empathy, and patience in our answers demands a considerable degree of active community building among the volunteer physicians. Key administrators are easily approachable for debriefing and conflict resolution among member physicians. In addition, a closed forum is used to discuss news and challenging questions and for a more unrestricted and informal conversation; this creates a safe space for critical feedback. The closed forum and personal messages among the volunteer physicians contribute to a supportive peer interaction and foster genuine interpersonal connections. We have witnessed the evolution of close relationships between physicians, even though they have never met.

The quality of the answers provided in the group is ensured largely by the community of the volunteer physicians and the approximately 30,000 daily active users. All answers are provided by named physicians, and supervision is accessible at all hours in the closed forum. Furthermore, physicians read one another’s answers and users are quick to notice errors or inconsistencies in the answers. Lastly, other professionals such as pharmacists, nurses, and engineers have contributed with nuance or correction to answers related to their respective fields. This informal web of quality control has proven to ensure a generally high quality, although this has not been formally validated.

Volunteer physicians were recruited mainly via already established Facebook communities for only physicians with Danish authorization and through snowballing recruitment. Once the workflow was established and the legal implications were clarified, we had a running proof of concept. This, in turn, made recruitment of doctors easier as many of them were hesitant to participate before seeing a working setup. We recruited physicians with all experience levels and specialties. All were subsequently verified through the Danish Authorization Registry and internal Facebook relations with other physicians. We have mobilized not only working physicians, but also those excluded from contributing to the COVID-19 workforce owing to sick leave, maternity or paternity leave, pregnancy, or quarantine, all of whom worked from home on their own conditions. This provides an indispensable workforce for the group and is meaningful to physicians marginalized in this crisis.

Users were recruited through sheer diffusion. We do not have any control over the outreach of our group. We initially shared our group in our personal networks, and the group has naturally grown since then. Some answers have been shared in public and private networks, in closed groups, or on Twitter, and some have even been shared by Instagram influencers, thus expanding the awareness of the group and the service we provide. This positively reflects the degree of trustworthiness our group has achieved; however, this also highlights a point of consideration among all physicians that for all answers they provide, they should withstand publication out of context.

Especially in the beginning of the COVID-19 pandemic, a large proportion of the questioners presented with insufficient eHealth literacy to navigate the information environment of the pandemic [13]. Answers to many questions are accessible on public information websites, but the ability to obtain proper and updated information and to apply the guidelines for activities of daily life has been inadequate. As the pandemic has progressed, the difficulty level of questions has risen, which suggests that public information sources have succeeded in further disseminating basic information on the novel coronavirus among the general public.

At the other end of the spectrum, we noticed that many questioners show a high degree of health and science literacy. Their questions on complex preliminary scientific discoveries are embellished with distortionary “clickbait” headlines, indicating their limitations in interpreting such complex data. Such diversity in the questions underlines a need for physicians of different backgrounds to answer different questions in different tones and temperaments. Consequently, the challenge of maintaining consistency in the answers coexists with the large diversity in the group of physicians.

## Future Perspectives

The Facebook group, “Spørg en Læge om Coronavirus,” was created to counteract misinformation and foster a feeling of safety during a stressful time. The group has received no funding; nonetheless, 57,000 unique members have joined, which indicates a need for this type of health service. This group demonstrates classic one-to-one counseling in combination with one-to-many communication where answers are visible to all members, thereby illustrating the current discourse of health communication. Currently, a news article can stimulate a personal discussion in the comments section (one-to-many communication becoming one-to-one communication), while a recording of an individual consultation can be distributed on social media platforms (one-to-one communication becoming one-to-many communication). One can thus argue that we here illustrate a future health communication premise that awaits almost every physician.

This group provides a proof of concept of a new way for health professionals to communicate and interact with the general public on social media platforms. The group is inspired by other Facebook groups and provides a template replicable in other similar or related initiatives. The Faroe Islands have already created the group, “Spyr ein Lækna um Korona,” which emulates the original initiative [14].

Our growing experience provides unique insights into the potential of Facebook in health communication; however, we cannot ignore the possibility of the distinctive information-seeking environment of the COVID-19 pandemic providing a favorable foundation for dissemination and upscaling of information. The extent and the validity of research on a health professional–driven social media platform should be further explored, preferably through a multidisciplinary approach and with an array of methodologies. Such studies could investigate developments in behavior, eHealth literacy, professional identity, and impact on physician-patient-relationships. Such a platform could also provide insights into patient adherence, navigation of health services, experiences with new treatments, management of common and rare diseases, and peer collaboration and communication. Therefore, further studies in this area would be highly relevant, and insights from this group should be further explored.

On World Patient Safety Day (September 17, 2020), the group "Spørg en læge om coronavirus” received the Danish Patient Safety Award 2020 in recognition of its effort to promote health understanding and a sense of safety through knowledge. This group was never intended to be a research study; the research potential was only recognized months after its launch. This group is part of a spontaneous health professional–driven emergency response at a unique time point where all community members have stepped up to contribute to their community in any way they can. This would limit the research potential of the data but adds a distinctive value of authenticity.
